# Empirical validation of photon recollision probability in single crowns of tree seedlings

**DOI:** 10.1016/j.isprsjprs.2020.08.027

**Published:** 2020-11

**Authors:** Aarne Hovi, Petri Forsström, Giulia Ghielmetti, Michael E. Schaepman, Miina Rautiainen

**Affiliations:** a Aalto University, School of Engineering, Department of Built Environment, P.O. Box 14100, FI-00076 Aalto, Finland; b University of Zürich, Department of Geography, Remote Sensing Laboratories, Winterthurerstrasse 190, CH–8057 Zurich, Switzerland; c Aalto University, School of Electrical Engineering, Department of Electronics and Nanoengineering, P.O. Box 15500, FI-00076 Aalto, Finland

**Keywords:** Multiangular, Reflectance model, Radiative transfer modeling, Spectral invariants, *p*-theory, Escape probability

## Abstract

Physically-based methods in remote sensing provide benefits over statistical approaches in monitoring biophysical characteristics of vegetation. However, physically-based models still demand large computational resources and often require rather detailed informative priors on various aspects of vegetation and atmospheric status. Spectral invariants and photon recollision probability theories provide a solid theoretical framework for developing relatively simple models of forest canopy reflectance. Empirical validation of these theories is, however, scarce. Here we present results of a first empirical validation of a model based on photon recollision probability at the level of individual trees. Multiangular spectra of pine, spruce, and oak tree seedlings (height = 0.38–0.7 m) were measured using a goniometer, and tree hemispherical reflectance was derived from those measurements. We evaluated the agreement between modeled and measured tree reflectance. The model predicted the spectral signatures of the tree seedlings in the wavelength range between 400 and 2300 nm well, with wavelength-specific bias between −0.048 and 0.034 in reflectance units. In relative terms, the model errors were the smallest in the near-infrared (relative RMSE up to 4%, 7%, and 4% for pine, spruce, and oak seedlings, respectively) and the largest in the visible wavelength region (relative RMSE up to 34%, 20%, and 60%). The errors in the visible region could be partly attributed to wavelength-dependent directional scattering properties of the leaves. Including woody parts of tree seedlings in the model improved the results by reducing the relative RMSE by up to 10% depending on species and wavelength. Spectrally invariant model parameters, i.e. total and directional escape probabilities, depended on spherically averaged silhouette to total area ratio (STAR) of the tree seedlings. Overall, the modeled and measured tree reflectance mainly agreed within measurement uncertainties, but the results indicate that the assumption of isotropic scattering by the leaves can result in large errors in the visible wavelength region for some tree species. Our results help increasing the confidence when using photon recollision probability and spectral invariants -based models to interpret satellite images, but they also lead to an improved understanding of the assumptions and limitations of these theories.

## 1 Introduction

Physically-based models in remote sensing are increasingly important in monitoring biophysical characteristics of vegetation. Such models are appropriate for many large-scale ecological applications, where it is often not feasible to obtain empirical training data that would sufficiently cover the spatial and temporal domains and measurement configurations. Examples of such applications are monitoring global canopy leaf area index (LAI) (e.g. Knyazikhin et al., 1998) or background reflectivity of canopies (Pisek and Chen, 2009). Vegetation in the physical models is parameterized using mathematical descriptions of canopy two- or three-dimensional structure together with spectral properties of the plant elements and background. Mathematical formulations of radiative transfer in canopies range from the turbid medium approach (e.g. Verhoef, 1984) to geometric-optical (e.g. Li and Strahler, 1985, Gerard and North, 1997) and hybrid models (e.g. Kuusk and Nilson, 2000, Lacaze and Roujean, 2001, Chen and Leblanc, 1997). The main difference between the approaches is the degree of detail in how vegetation structure is described at different hierarchical levels. Comprehensive comparisons of physically-based vegetation reflectance models have been regularly performed and documented (e.g. Widlowski et al., 2015).

Despite their benefits, physically-based approaches are still computationally resource intensive and often require a great variety and range of input data on vegetation structure. Although the former limitation is gradually reducing due to increasing computing power, the latter continues to be of particular relevance: it is practically difficult to obtain realistic information on (globally) representative spatial and temporal distributions of the input parameters, and large number of parameters may increase the ill-posed nature of the inversion problem (Baret and Buis, 2008). Thus, a remaining challenge is to develop simple, yet physically-based reflectance models which can simulate canopy reflectance with sufficient accuracy and handle uncertainties related to the radiation measurements. A potential theoretical framework for developing relatively simple models of forest canopy radiation regime is provided by the spectral invariants and photon recollision probability theories (Knyazikhin et al., 2011, Stenberg et al., 2016). According to these theories, the amount of radiation absorbed, reflected, or transmitted by a canopy depends on optical properties of foliage and one or more wavelength-independent (i.e. spectrally invariant) parameters describing canopy structure. In the simplest form, a model based on photon recollision probability theory takes as input only one wavelength-independent parameter, photon recollision probability (*p*), which can be interpreted as the “probability that a photon scattered from a leaf in the canopy will interact within the canopy again” (Smolander and Stenberg, 2005). Theoretical studies suggest that *p* is related to structure of coniferous shoots through silhouette to total area ratio (STAR) (Smolander and Stenberg, 2003), and to that of whole canopies through LAI and diffuse non-interceptance (Stenberg, 2007). These findings provide the necessary direct links between forest biophysical variables and the model parameters.

The theory of spectral invariants was presented roughly twenty years ago (Knyazikhin et al., 1998) and has been validated through simulations using e.g. Monte Carlo ray tracing (MCRT) (e.g. Disney and Lewis, 2007). However, empirical validations of the theory have been performed only for a few forest canopies (Panferov et al., 2001, Wang et al., 2003) and for individual shoots in laboratory conditions (Rautiainen et al., 2012). What is currently missing is an empirical validation at intermediate levels, such as individual tree crowns. Empirical validation at all hierarchical levels is important to evaluate the model performance in nature and to holistically understand the effects of different simplifying assumptions. Ultimately, this would help in developing more realistic models and reducing the uncertainties in retrievals of biophysical variables from remote sensing data.

This paper presents results of the first empirical validation of a model based on photon recollision probability at the level of individual tree crowns. We designed an experiment to measure multiangular reflectance patterns of small single trees. Based on their size (height = 0.38–0.7 m, stem diameter less than 2.5 cm, age up to 4 years), our study trees are classified as tree seedlings according to common terminology in forestry, but for the sake of conciseness, we will call them simply ‘trees’ in our theory, materials and methods, and results sections. We used the data to address the following research questions:

1) How accurately can a model based on photon recollision probability simulate directional scattering properties of individual tree seedlings?
2) How much does the model accuracy improve, if woody elements are taken into account in the simulation?
3) How are the spectrally invariant parameters of the model linked to tree seedling structure?

## 2 Theory

### 2.1 Overview

In this section, we review the theory and concepts that are used throughout our study. We start with concepts related to scattering of shortwave radiation by trees and their foliage (Section 2.2), continue by deriving the scattering model that we validate (Section 2.3), and finally review the concept of silhouette to total area ratio (STAR, Section 2.4). Symbols and abbreviations as used in our study are presented in Table 1.

**Table 1:** List of symbols and abbreviations.

Symbol/abbreviation	Explanation
*ω* (*ω_tree_*, *ω_leaf_*)	Albedo (of a tree, leaf) [unitless]
*ω*(Ω)	Directional scattering coefficient [sr^-1^]
*ω*(2π)	Hemispherical reflectance i.e. ‘half-albedo’ [unitless]
dΩ	Solid angle [sr]
Ω, Ω*_i_*	Direction of view, direction of illumination
*R*	Reflectance [unitless]
*T*	Transmittance [unitless]
*p*	Photon recollision probability [unitless]
*ρ*(Ω)	Directional escape probability [sr^-1^]
STAR	Spherically averaged silhouette to total area ratio [unitless]
$\overset{¯}{S}$	Spherically averaged silhouette area of a tree [m^2^]
*S_tree_*	Silhouette area of a tree [m^2^]
*S*_WR_*__tree_*	Surface area of a white reference panel [m^2^]
*L*	Total leaf area per tree [m^2^]
*θ*, *φ*	Zenith angle, azimuth angle [degrees]
*w_j_*	Weight of Gauss-Legendre integration (*j* denotes view zenith angle) [unitless]
WR	White reference
DN*_leaf_*, DN*_tree_*	Signal obtained from leaf (or needle) sample, or from a tree [digital numbers]
DN_WR_*__leaf_*, DN_WR_*__tree_*	Signal obtained from a white reference panel in leaf (or needle) measurements, or in tree measurements [digital numbers]
*R*_WR_*__leaf_*, *R*_WR_*__tree_*	Reflectance of the white reference panel used in leaf (or needle) measurements, or in tree measurements
*G*	Gap fraction in a needle sample
*E*	Irradiance [W m^−2^]
*k*_DN_	Efficiency of the spectrometer [DN W^−1^]
PSF	Point spread function
FOV	Field of view
*f_tree_*(Ω), *f*_WR_*__tree_*	Silhouette area of a tree (or surface area of a white reference panel) weighted by the PSF of the spectrometer [m^2^]
*s_ij_*	Discrete representation (i.e. a binary image) of the silhouette of tree or white reference panel: letters *i* and *j* refer to row and column, respectively
DN*_stray_*	Stray light signal when measuring empty goniometer (without tree or white reference panel) [digital numbers]
*b_tree_, b*_WR_*__tree_*	Fraction of stray light not obscured by the tree or white reference panel
DN*_total_*_,_*_tree_*, DN*_total_*_,WR_*__tree_*	Signal including stray light when measuring a tree, or white reference panel [digital numbers]
VNIR	Visible-near-infrared detector (350–1000 nm)
SWIR1	First shortwave-infrared detector (1001–1800 nm)
SWIR2	Second shortwave-infrared detector (1801–2500 nm)

### 2.2 Concepts

Albedo (*ω*, [unitless]) is the fraction of incoming radiation that is scattered (i.e. not absorbed) by an object. In statistical terms, it is the probability that a photon, after being intercepted by an object, will be scattered. The directional scattering coefficient [sr^-1^], denoted here as *ω*(Ω), gives the probability density of scattered photons (per steradian) observed in view direction Ω. Integration of *ω*(Ω) over all spherical directions gives the albedo

(1) $$\omega =\int_{4\pi }\omega \left(\Omega \right)d\Omega$$

Albedo is wavelength-dependent, but to simplify the notation the sign of wavelength (λ) is not included in our formulae. For an ideal isotropic scatterer, i.e. an object that does not absorb anything and scatters equally in all directions, *ω* equals 1 and *ω*(Ω) at any Ω equals 1/(4π) = 0.0796 sr^-1^.

Further, we define ‘half-albedo’ (i.e. *ω*(2*π*)) as fraction of incoming radiation that is scattered into a hemisphere. We can then define reflectance and transmittance as half-albedo measured in two opposite hemispheres, separated by a reference plane. Albedo equals the sum of reflectance and transmittance. We use these definitions of reflectance and transmittance throughout the study. For leaves and needles of trees, we define the reference plane as being perpendicular to the leaf normal. Thus, in case of normal incidence, the radiation that is scattered at phase angles smaller than 90° constitutes reflectance, and radiation that is scattered at phase angles larger than 90° constitutes transmittance. Further, in our scattering model (Section 2.3) we assumed that the leaf reflectance and transmittance were independent of the angle between leaf normal and direction of incoming radiation. For trees, we define the reference plane as being parallel to the Earth surface. In practice (see Section 3), we measured reflectance and transmittance of leaves and needles with integrating spheres (near-normal illumination), and computed reflectance of trees from estimates of *ω*(Ω), derived from multiangular measurements made in a goniometer applying a bi-conical view-illumination geometry. We denote albedo of leaves (or needles) and trees by symbols *ω_leaf_* and *ω_tree_*, respectively.

### 2.3 Scattering model of a tree

The model that we validate in our study is based on the concept of photon recollision probability (see review by Stenberg et al. (2016) and references therein). We present its derivation here for sake of thoroughness, because the exact formulations vary from one study to another, depending e.g. on whether individual shoots or whole forest canopies are modeled. Note that the model has not been empirically validated for individual trees previously.

The probability that a photon will be scattered after being intercepted by a tree is equal to leaf albedo (*ω_leaf_*). Leaf is assumed to scatter isotropically. The probability density of photons escaping from the tree crown in direction Ω is called the directional escape probability (*ρ*(Ω), [sr^-1^]). Thus, the probability that a photon, after being intercepted, will be scattered once and will then escape in direction Ω without any further interactions is

(2) $$\omega_{\mathrm{tree},1}\left(\Omega \right)=\omega_{\mathrm{leaf}}\rho \left(\Omega \right)$$

On the other hand, the probability that the photon would be re-intercepted after scattering is determined by photon recollision probability (*p*). Thus, the probability that a photon will be scattered twice and will then escape in direction Ω is

(3) $$\omega_{\mathrm{tree},2}\left(\Omega \right)=\omega_{\mathrm{leaf}}p\omega_{\mathrm{leaf}}\rho \left(\Omega \right)$$

We can further continue to third and fourth scattering event, and so on. The total probability that a photon, after being intercepted by a tree, will escape in direction Ω is

(4) $$\omega_{\mathrm{tree}}\left(\Omega \right)=\omega_{\mathrm{leaf}}\rho \left(\Omega \right)+\omega_{\mathrm{leaf}}p\omega_{\mathrm{leaf}}\rho \left(\Omega \right)+\omega_{\mathrm{leaf}}p\omega_{\mathrm{leaf}}p\omega_{\mathrm{leaf}}\rho \left(\Omega \right)+...+\omega_{\mathrm{leaf}}{\left(p\omega_{\mathrm{leaf}}\right)}^{n-1}\rho \left(\Omega \right)+...$$

where *n* is the number of scattering event, often also called ‘order of scattering’. Eq. (4) is a descending geometric series with common ratio *ω_leaf_ρ*(Ω). The sum of the series is

(5) $$\omega_{\mathrm{tree}}\left(\Omega \right)=\rho \left(\Omega \right)\frac{\omega_{\mathrm{leaf}}}{1-p\omega_{\mathrm{leaf}}}$$

This is the model that we will be validating in our experiment. In Eq. (5), it is assumed that the photon recollision and escape probabilities do not depend on the order of scattering. Eq. (5) can be rearranged in the form

(6) $$\frac{\omega_{\mathrm{tree}}\left(\Omega \right)}{\omega_{\mathrm{leaf}}}=\omega_{\mathrm{tree}}\left(\Omega \right)p+\rho \left(\Omega \right)$$

from which it is possible to retrieve the values of spectrally invariant parameters *p* and *ρ*(Ω), if *ω_tree_*(Ω) and *ω_leaf_* are known. This is done by plotting *ω_tree_*(Ω)/*ω_leaf_* against *ω_tree_*(Ω) in two or more wavelengths, and fitting a regression line into the observations (Schull et al., 2011, Knyazikhin et al., 2013). The values of *p* and *ρ*(Ω) are obtained as the slope and intercept of the regression line. The possibility to retrieve model parameters directly from measurement data was important, because while analytical solution for *p* in coniferous shoots (potentially also applicable to trees) exists (Smolander and Stenberg, 2003) and also formulae for the directional scattering by trees with random leaf spatial distribution have been presented (Dickinson et al., 2008, Dickinson, 2008), an exact computation of *ρ*(Ω) for trees of arbitrary (a priori unknown) leaf spatial distribution is not possible. Thus, retrieval of *ρ*(Ω) from our data enabled us to focus in this paper on evaluating the validity of the model itself and its assumption of spectral invariance, rather than uncertainties in estimating *ρ*(Ω).

### 2.4 Silhouette to total area ratio (STAR)

The concept of silhouette area to total leaf (or needle) area ratio (STAR) originates from ecological studies, where it was used for quantifying the degree of self-shading and light interception efficiency of coniferous shoots (Norman and Jarvis, 1974, Carter and Smith, 1985). Later, to facilitate comparisons between studies, it became common to use the spherically averaged STAR (Oker-Blom and Smolander, 1988). In the field of remote sensing, Smolander and Stenberg (2003) showed that 4 × STAR can be also interpreted as “probability of no interaction”, and therefore photon recollision probability of coniferous shoots is related to STAR as *p* = 1–4 × STAR (Section 2.4. in Smolander and Stenberg, 2003). Empirical proof was later presented by Rautiainen et al. (2012) who measured albedo of Scots pine shoots in a similar goniometer setting compared to our experiment. They found that shoot albedo, that was upscaled from needle albedo using similar model as we presented in Section 2.3 combined with *p* values derived from STAR, agreed with measurements, relative RMSE being up to ~ 12%. There are, however, only few simulation studies that have looked at STAR of entire trees: Stenberg et al. (2014) examined dependence of STAR on tree size and forest density, and Wang et al. (2020) examined its effect on tree scattering. Wang et al. (2020) showed a strong dependence between *p* and 1–4 × STAR for modeled tree crowns. Further, STAR might be related to the directionality of scattering (i.e. *ρ*(Ω)), as shown for coniferous shoots (Section 3 in Rautianen et al., 2018). Throughout the current study, we use spherically averaged STAR, defined as

(7) $$\text{STAR}=\frac{\overset{¯}{S}}{L}=\frac{1}{L}\frac{1}{4\pi }\int_{4\pi }S_{\mathrm{tree}}\left(\Omega \right)d\Omega$$

where $\overset{¯}{S}$is the spherically averaged silhouette area, *L* is total leaf (or needle) area in the tree, and *S_tree_*(Ω) is the silhouette area of the tree in direction Ω.

## 3 Materials and methods

### 3.1 Sample tree seedlings and measurement protocol

We measured a total of 18 seedling trees during Aug–Sept 2018, using a laboratory goniometer at the Remote Sensing Laboratories, University of Zürich (Dangel et al., 2003, Mõttus et al., 2012). The aim was to estimate *ω_tree_*(Ω) at different view directions over the hemisphere from the measurements made in the goniometer, and to determine leaf (or needle) and bark albedo and STAR of each tree. To be able to analyze the effect of woody parts, STAR was computed both by including and excluding woody parts in total area calculation. These measurements were then used to validate the model presented in Section 2.3. The trees represented different species (Scots pine (*Pinus sylvestris* L.), Norway spruce (*Picea abies* (L.) H. Karst), sessile oak (*Quercus petraea* (Matt.) Liebl.), and were between 0.38 m and 0.7 m in height. Trees with visually different crown structure were selected on purpose, to allow for maximum variation in STAR, in order to examine the relations between STAR and scattering induced by the tree. The trees were brought from a local nursery. We stored the trees outside and watered them frequently to avoid water stress. All trees were grown in standardized pots and had no visual damage effects. Immediately before the measurement, a tree was brought indoors to the laboratory.

We will describe the measurements and data processing in the following sections 3.2–3.6. The measurements will be presented in chronological order, and will be accompanied with the descriptions of data processing. The only exception is the data processing to derive *ω_tree_*(Ω) from the goniometer measurements, which will be presented last (Section 3.6), because it required inputs from several measurements. Model validation procedure will be elaborated on in Section 3.7.

### 3.2 Measurements of multiangular spectra of tree seedlings in the goniometer

**Fig. 1:**
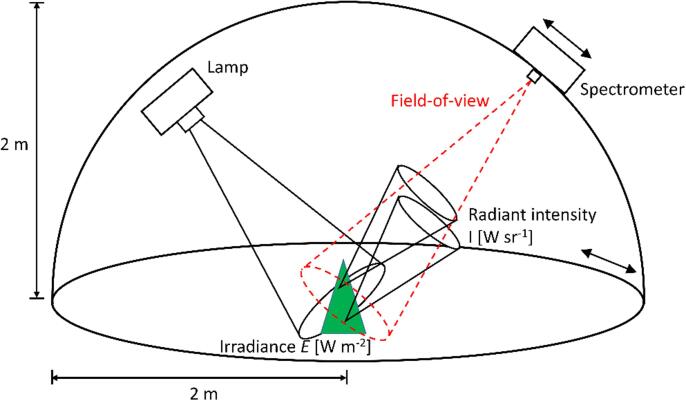
Illustration of the measurement principle of the laboratory goniometer setup. The tree was placed in the middle. The supporting structures (e.g. the pot where the tree was grown, and the wooden frame that was used to minimize stray light) are ignored in this illustration for clarity. The tree was illuminated with a conical light beam that had an irradiance *E* when it reached the center of the tree. Each scattering object within the tree produced a radiant intensity *I* towards the spectrometer, which then measured an average *I* within its field-of-view.

During the measurements, each tree was placed such that the center of its crown coincided with the center point of the goniometer (Fig. 1). The detector unit of the spectrometer, i.e. a bare fiber-optic bundle, located at a 2 m distance from the center point, was aligned to point exactly at the center of the tree. The nominal field of view (FOV) of the bundle had an opening angle of 25°. The spectrometer was an ASD FieldSpec3 (serial nr 16006), and it measured at 1 nm intervals between 350 and 2500 nm. The spectrometer is regularly calibrated, traceable to a secondary traceable reference standard, with raw data, radiance and reflectance calibration methods (Schaepman and Dangel, 2000, Milton et al., 2009). The illumination source was a 1000 W brightness stabilized tungsten halogen lamp that generated a conical light beam using a Köhler illuminator with aspherical reflector and a condenser. The light beam had an opening angle of approx. 22° (c.f. Rautiainen et al., 2012 for details). The illumination zenith angle was 40° and the lamp was at a 1.75 m distance from the center of the goniometer. The sizes of the measured trees (max. height 0.7 m) were selected so that the tree was always fully illuminated by the lamp.

A white reference measurement was made before and after measurement of each tree. We used a 20 × 20 cm calibrated Zenith Lite® panel that had nominal reflectance of 95%. It was placed on a tripod at the center of the goniometer. The white reference measurement was always made at nadir. The measurements of a tree were made at 12 equally-spaced azimuth directions (*φ* = 15° + *n* × 30°), and at three zenith angles per azimuth (*θ* = [21.2°, 48.6°, 76.2°]). The zenith angles were chosen so that the reflectance of the tree could be calculated from the measurements, using Gauss-Legendre integration so that cos*θ* correspond to the Gauss-Legendre nodes. The same zenith angles were used also by Mõttus et al., 2012, Rautiainen et al., 2012 who measured scattering phase functions of coniferous shoots using the same goniometer. In addition, the nadir direction (*θ* = 0°) was measured once for every other azimuth. Measurements in the cross plane (*φ* = 90°) and in the principal plane (*φ* = 0°) excluding directions behind the lamp were also made, but were not used in the current study, because we focused on hemispherically integrated reflectance and thus preferred observations with equal angular spacing.

To minimize stray light, a special table (1.5 × 1.3 m frame built of wood) was used in the measurements. It was covered with a black cloth with directional hemispherical reflectance of approx. 2% in all measured wavelengths. The cloth had an opening, which allowed the tree to be placed so that the pot in which the tree was growing was under the cloth, and only the tree crown was above the cloth. Similarly, the tripod holding the white reference panel was covered by the cloth when measuring white reference. Any radiation from the light beam that did not hit the tree (or white reference) was therefore absorbed and only marginal amounts were reflected by the black cloth. The height of the table was adjustable to ensure that it could be set approximately at the level of the base of the tree, independent of the tree height. In practice, we used four predefined heights (0.6 m, 0.65 m, 0.69 m, and 0.74 m) to facilitate stray light corrections. To quantify the amount of stray light, the cloth-covered table without a tree was measured following the same measurement protocol as for the tree measurements. The stray light measurement was repeated for all table heights and it allowed to express stray light as a fraction of white reference signal. This meant that stray light needed to be measured only once per each table height, which notably reduced the overall time needed for measurements.

### 3.3 Silhouette area photography and data processing

After recording the last white reference, the tree was placed back in the spectrometer FOV, and silhouette photographs were taken from the same view angles as the spectral measurements. We used a digital camera (Nikon D5000), which had an adjustable focal length fixed at 45 mm. The camera was attached to the goniometer, close to the detector unit of the spectrometer. The viewing geometry was therefore almost the same as with the spectrometer (difference of ~ 3° due to ~ 10 cm displacement of the camera). In addition, one photograph was taken exactly from the direction of illumination while the position of the lamp was lowered to allow the camera to see the tree. The camera was calibrated for interior orientation parameters (focal length, principal point, lens distortion parameters) by taking images of a checkerboard pattern placed on a table in the center of the goniometer. The same checkerboard pattern was used for solving the exterior orientation parameters, i.e., the camera locations and orientations. We used Matlab Computer Vision Toolbox^TM^ for camera calibration and orientation. When taking the photographs, the tree was illuminated with two LED lights from the sides to reduce shadows on a white canvas that was placed behind the tree. We used f-number of *f*/8 and adjusted exposure time manually so that white background was slightly underexposed (by 0.7 exposure stops according to the camera built-in light meter). This was done in order to avoid overexposure in any of the photographs. In addition, we used the autobracketing function of the camera to take images with exposure times adjusted by 0.7 exposure stops downwards and upwards, to evaluate the effect of exposure time on our results.

For each image, an area of image that contained the tree was manually delineated. We then applied an automatic thresholding algorithm (Otsu 1979) to the blue channel of the images, to separate the tree from the white background. The distance of the camera and focal length were used to calculate pixel size at the center of the goniometer, and silhouette area was computed as the sum of pixels belonging to the tree times the pixel area. All parts of the tree were thus assumed to be at a 2 m distance from the camera, which results in small inaccuracies due to parallax errors. However, this was unavoidable because the camera could not be placed any further from the target. Finally, the spherically averaged silhouette area of each tree was computed as

(8) $$\overset{¯}{S}=\frac{2\pi }{12}\sum_{i=1}^{12}\sum_{j=1}^{3}w_{j}S_{\mathrm{tree}}\left(\Omega_{\mathrm{ij}}\right)$$

where *i* and *j* are the azimuth and zenith angles, respectively, and *w_j_* are the weights of Gauss-Legendre integration, normalized so that sum of weights per each azimuth equals 1. Division by 12 comes from the fact that we had 12 azimuth angles. The silhouette area of tree in the direction of illumination was not used here, but was used in processing the spectral measurement data (Section 3.6).

### 3.4 Measurements and processing of leaf and bark spectra

After silhouette area photography of each tree, we randomly picked three leaves, or three samples of needles from the tree, and measured reflectance and transmittance of both sides of each sample. In conifers, all needle age classes were mixed randomly. The samples were measured with an ASD RTS-3ZC integrating sphere attached to an ASD FieldSpec3 spectrometer (serial nr 16007) (Yáñez-Rausell et al., 2014a, Yáñez-Rausell et al., 2014b). We used spectrally black needle carriers (see e.g. Hovi et al., 2020) of 0.8 mm thickness and placed needles at 0.5–1 needle widths apart. Also the leaves of broadleaved trees were placed in the needle carriers to ensure a comparable measurement. Spruce needles were short and therefore they were placed in the carrier in two rows, so that the light beam illuminated the tips of the needles. For pine, measurements close to the center of needles were made, and for oak a randomly selected spot on the leaf was measured so that major veins in the leaf were avoided. The measurement protocol was the same as used by Hovi et al. (2020). An uncalibrated Spectralon® panel was used as white reference and was calibrated afterwards against a known standard.

Reflectance (*R_leaf_*) and transmittance (*T_leaf_*) were computed as

(9) $$R_{\mathrm{leaf}}=\frac{\text{D}{\text{N}}_{\mathrm{leaf},R}}{\text{D}{\text{N}}_{\text{WR}\_leaf,R}}\frac{1}{1-G_{R}}R_{\text{WR}\_leaf}$$

and

(10) $$T_{\mathrm{leaf}}=\frac{\text{D}{\text{N}}_{\mathrm{leaf},T}-G_{T}}{\text{D}{\text{N}}_{\text{WR}\_leaf\text{,}T}}\frac{1}{1-G_{T}}R_{\text{WR}\_leaf}$$

where DN*_leaf_* and DN_WR_*__leaf_* are the readings taken from the sample and white reference, respectively, *R*_WR_*__leaf_* is the reflectance of the white reference, and *G_R_* and *G_T_* are the gap fractions in the sample (slightly different for reflectance and transmittance measurements due to different measurement geometry). For oak leaves, gap fractions were zero. Gap fraction in a needle sample was obtained by scanning the needle carrier with needles in it, using a digital film scanner (Epson Perfection V550), and by applying a threshold to the obtained 8-bit grayscale images to separate needles from the background (Hovi et al., 2020). For needles of pine and spruce, the threshold value (202 for pine, 187 for spruce) was optimized by forcing the needle transmittance to predetermined values at 410–420 nm. In this region, needle transmittance is close to zero with small residual variation depending on the sample, and thus the errors of the estimated gap fraction due to assuming constant transmittance are minimized. Yet the signal-to-noise ratio of the spectrometer at those wavelengths is acceptable compared to wavelengths lower than 410 nm. The transmittance values for pine and spruce needles at 410–420 nm were obtained in a separate measurement campaign in 2019, for the same species but grown in Finland, and were 0.021 ± 0.007 (mean ± standard deviation) for pine and 0.039 ± 0.007 for spruce. In that campaign, the gap fractions in the needle samples were obtained by painting the illuminated side of needles in black, which prevented specular reflection from the illuminated side to contribute to transmittance and thus the observed transmittance was purely caused by the light transmitted through the gaps in the needle sample (Daughtry et al., 1989). Finally, we applied an empirical bias correction to all processed leaf and needle transmittance spectra (adjusting transmittance 5.5% downwards) that was taken from the measurements made against a trusted reference method in Hovi et al. (2020). This ensured that leaf and needle albedo did not exceed unity in any of the measurements.

Reflectance spectra of tree bark were measured for three sample trees (one per species). Three bark samples were carefully peeled from the tree trunk. Each sample was placed in the needle carrier, and its reflectance spectrum was measured. The measurement protocol and processing were similar as for leaves. Bark albedo was assumed equal to bark reflectance in the spectral modeling. Leaf reflectance and transmittance spectra were summed to yield leaf albedo. Finally, leaf and bark albedo spectra were smoothed with a Savitzky-Golay filter (Savitzky and Golay, 1964) to remove the high-frequency noise that was present, particularly affecting lower and upper wavelengths of the spectra, i.e. (near-)ultraviolet and shortwave-infrared regions.

### 3.5 Measurements and processing of leaf and woody areas

Total leaf area for each tree was determined by destructive measurements. A subset of leaves or needles from each tree was scanned and weighed for determining the ratio of projected area to leaf or needle (fresh) mass. The sample sizes for spruce and pine were 1 g and 10 g (approximately 150 needles), respectively, and that of oak was 5 g. In preliminary tests, these sizes were determined sufficient to result in negligible standard errors. For conifers, an additional small subset (10 needles) was taken for measurements of needle length and two widths. The width measurements were made in the middle between the tip and base of each needle, corresponding to the breadth and thickness of the almost half-cylinder-shaped cross section of the pine needles, and two transverse dimensions of the diamond-shaped cross section of the spruce needles. This subset was used for converting projected area to total area. In order to compute the total needle area from the measurements of needle dimensions, the shape of spruce needles was assumed as parallelepiped (Eq. (9) in Sellin, 2000), and that of pine needles as semi-fusiform (Eq. (7) in Flower-Ellis and Olsson, 1993). For oaks, the total area of a leaf was two times the projected area. Finally, all leaves or needles from the tree were picked and weighed, and the total leaf area of a tree was calculated based on leaf (or needle) mass and the above determined linear conversion factors: mass to projected area [m^2^ g^−1^] and projected to total area [m^2^ m^−2^].

For computing the woody area of the trees, silhouette photographs of the trees with leaves removed were taken. The photography of trees without leaves was otherwise similar to the photography with leaves (Section 3.3), but only every 3rd azimuth angle was used in order to save time in the laboratory work. Also the data processing followed the same procedure (Section 3.3), with the only exception that some of the images needed to be manually edited, i.e. areas erroneously detected as tree were painted in white, because the automatic thresholding did not work perfectly for the trees without leaves. The total area of woody parts for each tree was computed from the spherically averaged silhouette area, assuming that the total woody area equals four times the spherically averaged silhouette area, which is true for any convex body (Lang, 1991). The woody parts were very sparse, so this is a realistic assumption, unlike for leaves, and particularly needles, that can be highly clumped and thus self-shadowed.

### 3.6 Deriving directional scattering coefficients of the tree seedlings from goniometer measurements

#### 3.6.1 Measurement equations

Because the tree was treated as a point-like scatterer (as opposed to a surface) in our model, we did not use simple normalization by a white reference panel to compute bidirectional reflectance factor (BRF), but rather aimed at deriving an estimate of *ω_tree_*(Ω) [sr^-1^] from goniometer measurements. This was done by writing the equations that mathematically describe the signals recorded by the spectrometer during the measurements of white reference panel and the tree, and then solving for *ω_tree_*(Ω). The equation that models the signal [digital numbers] recorded by the spectrometer for the white reference panel at nadir is

(11) $$\text{D}{\text{N}}_{\text{WR}\_tree}=ES_{\text{WR}\_tree}\cos40^{{}^{\circ}}\frac{R_{\text{WR}\_tree}\cos0^{{}^{\circ}}}{\pi }d\Omega k_{\text{DN}}$$

where *E* is the irradiance of the lamp at the center of the goniometer [W m^−2^]. Part of the irradiance is intercepted by the white reference panel. The surface area of the panel is *S*_WR_*__tree_* [m^2^] and the projected area towards the direction of illumination is *S*_WR_*__tree_*cos40°. Multiplication of *E* by *S*_WR_*__tree_*cos40° gives the radiation intercepted by the panel [W]. The result is multiplied by the directional scattering coefficient of the panel at nadir [sr^-1^], to obtain the radiant intensity towards the sensor [W sr^-1^]. Here it is assumed that the white reference panel is an ideal Lambertian surface, and thus its directional scattering coefficient is *R*_WR_*__tree_*cos*θ*/*π*. To obtain the radiation that enters the detector [W], the radiant intensity is multiplied by the solid angle subtended by the detector (*d*Ω, [sr]). The resulting radiant flux [W] is further multiplied by the ‘efficiency’ of the spectrometer (*k*_DN_, [DN W^−1^]). Note that in these computations it is assumed that the response of the spectrometer is linear and zero-DN means zero photons, i.e. dark current and stray light have been subtracted.

Similarly, the measurement equation for the signal recorded for the tree at direction Ω is

(12) $$\text{D}{\text{N}}_{\mathrm{tree}}\left(\Omega \right)=ES_{\mathrm{tree}}\left(\Omega_{i}\right)\omega_{\mathrm{tree}}\left(\Omega \right)d\Omega k_{\text{DN}}$$

where *ω_tree_*(Ω) is the directional scattering coefficient of the tree [sr^-1^] in direction Ω, and *S_tree_*(Ω_i_) is the silhouette area of the tree in the direction of illumination [m^2^].

We can solve *ω_tree_*(Ω) from Eqs. (11), (12) as

(13) $$\omega_{\mathrm{tree}}\left(\Omega \right)=\frac{\text{D}{\text{N}}_{\mathrm{tree}}\left(\Omega \right)}{\text{D}{\text{N}}_{\text{WR}\_tree}}\frac{S_{\text{WR}\_tree}\cos40^{{}^{\circ}}}{S_{\mathrm{tree}}\left(\Omega_{i}\right)}\frac{R_{\text{WR}\_tree}\cos0^{{}^{\circ}}}{\pi }$$

To take into account the spatially varying response of the detector of the spectrometer, we introduced a correction factor *f*_WR_*__tree_*/*f_tree_*(Ω), which takes into account that the tree and white reference panel can be located in slightly different areas within the FOV of the detector, and the observed signal depends on the sensitivity of the detector in these areas, i.e. the point spread function (PSF). Factors *f*_WR_*__tree_* and *f_tree_*(Ω) were obtained as the surface area of the white reference panel (*S*_WR_*__tree_*) and silhouette area of the tree in the view direction (*S_tree_*(Ω)), respectively, weighted by the PSF of the detector. The ratio of these factors becomes larger when the tree is located more towards the edges of the PSF compared to the white reference panel. The final equation for *ω_tree_*(Ω) then becomes

(14) $$\omega_{\mathrm{tree}}\left(\Omega \right)=\frac{\text{D}{\text{N}}_{\mathrm{tree}}\left(\Omega \right)}{\text{D}{\text{N}}_{\text{WR}\_tree}}\frac{S_{\text{WR}\_tree}\cos40^{{}^{\circ}}}{S_{\mathrm{tree}}\left(\Omega_{i}\right)}\frac{R_{\text{WR}\_tree}\cos0^{{}^{\circ}}}{\pi }\frac{f_{\text{WR}\_tree}}{f_{\mathrm{tree}}\left(\Omega \right)}$$

#### 3.6.2 Correction for the point spread function of the spectrometer

**Fig. 2:**
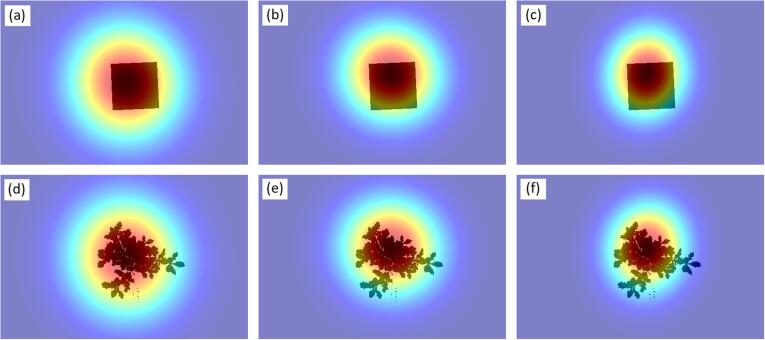
Point spread functions (PSF) of the three detectors of the spectrometer overlaid on the silhouette images of the white reference panel (upper row) and an oak tree (lower row). The PSFs were slightly different for each of the three detectors of the spectrometer: visible-near-infrared (a,d), shortwave-infrared 1 (b,e), and shortwave-infrared 2 (c,f). The examples here represent nadir view. In practice, the white reference panel was always viewed at nadir, but the viewing direction while measuring each tree varied. The dimensions of the white reference panel are 20 × 20 cm.

We modeled the PSF of the detector as an asymmetric 2D Gaussian function (Fig. 2). Its parameters were determined by fitting the function into measurements taken of a small 1.25-inch Spectralon panel that was placed on the surface of the black cloth and moved in the FOV of the detector, with the detector at nadir. Each time the panel had been moved into a new position, a reading with the spectrometer was taken. Stray light was also measured and subtracted from these measurements. The remaining signals were averaged over all bands in each detector, i.e. VNIR = 350–1000 nm, SWIR1 = 1001–1800 nm, or SWIR2 = 1801–2500 nm. Noisy regions below 400 nm and above 2300 nm were excluded. The average signal at each position was normalized to the average signal obtained from the center of the FOV, and the Gaussian function was fitted into these observations. The process resulted in three PSFs, one for each detector of the spectrometer (Fig. 2). Each of them was projected at the center of the goniometer and interpolated into a 0.5 × 0.5 mm grid. For each view direction, the interpolated PSF was rotated so that it was perpendicular to view direction, and was then projected onto the binarized silhouette images of the tree and the white reference panel (Fig. 2). Finally, *f*_WR__*_tree_* and *f_tree_*(Ω) were computed as

(15) $$f=\sum_{i=1}^{m}\sum_{j=1}^{n}\text{PS}{\text{F}}_{\mathrm{ij}}s_{\mathrm{ij}}$$

where *s* is the binarized silhouette image of either the tree or white reference panel. Applying *f*_WR_*__tree_*/*f_tree_*(Ω) reduced the discontinuities in the *ω_tree_*(Ω) spectra between the detectors. These ‘sensor jumps’ (mean ± standard deviation) were reduced from −9.5 ± 9% to −7 ± 7.9% (at 1001 vs. 1001 nm), and from −3 ± 3.9% to 0.8 ± 3.5% (at 1801 vs. 1800 nm), when expressed as relative to the mean *ω_tree_*(Ω) in these regions. The sensor jumps can be also due to varying temperature responses of the detectors (Hüeni and Bialek, 2017), but because we were operating indoors at a stable temperature, the remaining sensor jumps after correction are likely caused by the inaccuracies in determining the correction factor *f*_WR_*__tree_*/*f*_tree_(Ω) due to e.g. the fact that the illumination was not spatially evenly distributed in all parts of the tree crown.

#### 3.6.3 Stray light correction

**Fig. 3:**
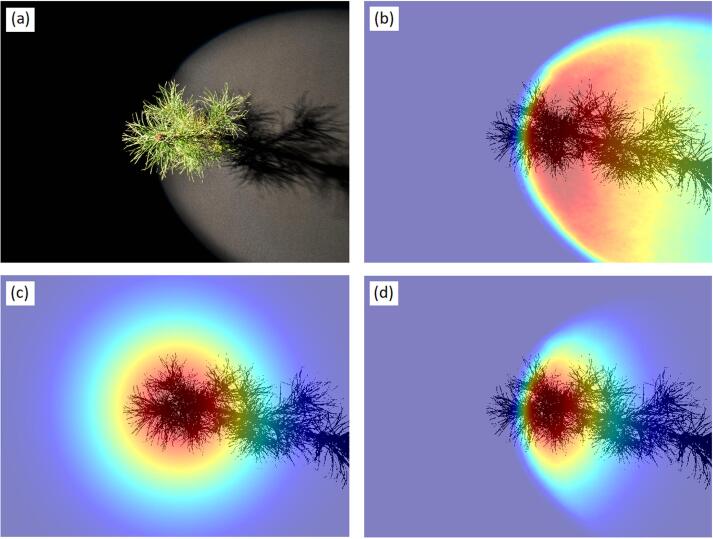
Illustration of the computation of *b_tree_*, i.e. the fraction of stray light that remained in the presence of tree in the goniometer measurements (view azimuth 165°, view-zenith 21.2°, light originates from west-northwest direction). Sub-figure (a) shows a pine tree illuminated in the goniometer. Subfigure (b) illustrates the irradiance distribution of the light beam that was modeled from the red channel of RGB images taken of the light beam and linearized (i.e. gamma correction removed), (c) the point spread function (PSF) of the spectrometer in VNIR detector, and (d) the irradiance of the light beam weighted by the PSF of the spectrometer (‘PSF-weighted irradiance’). An estimate of *b_tree_* was obtained by computing the sum of PSF-weighted irradiance in those areas that were not covered by the tree and its shadow, and ratioing the result to the total sum of PSF-weighted irradiance. The silhouette and shadow of the tree shown in sub-figures (b)–(d) were obtained from the silhouette area photographs (Section 3.3).

As mentioned earlier, stray light had to be removed from the spectrometer measurements before using Eq. (14). Because the stray light fraction was known from measurements, stray light [digital numbers] could be calculated for any view angle based on a measurement of the white reference panel. The challenge was that the tree (or white reference panel) and its shadow obscured some fraction of the stray light. Therefore, for an accurate stray light removal, we used the formulae

(16) $$\text{D}{\text{N}}_{\mathrm{tree}}=\text{D}{\text{N}}_{\mathrm{total},tree}-b_{\mathrm{tree}}\text{D}{\text{N}}_{\mathrm{stray}}$$

and

(17) $$\text{D}{\text{N}}_{\text{WR\_}tree}=\text{D}{\text{N}}_{\mathrm{total},\text{WR\_}tree}-b_{\text{WR\_}tree}\text{D}{\text{N}}_{\mathrm{stray}}$$

where DN*_tree_* and DN_WR_*__tree_* are the signals free from stay light, DN*_total,tree_* and DN*_total,_*_WR__*_tree_* are the original DN values observed, DN*_stray_* is the stray light signal without the presence of the tree or white reference panel, and *b_tree_* and *b*_WR__*_tree_* are the fractions of stray light that remain when the tree (or white reference panel) and its shadow are present. Calculations of *b_tree_* and *b*_WR__*_tree_* were done for each of the detectors of the spectrometer separately, utilizing the silhouette area photographs taken of the trees (or white reference panel), and is explained in Fig. 3 and its caption. Multiple scattering between the tree and the background was ignored in stray light corrections. However, multiple scattering mainly occurs in near-infrared, where the stray light fraction is small and uncertainty in stray light contributes little to the uncertainty of *ω_tree_*(Ω). Depending on the detector, the *b_tree_* (mean ± standard deviation) was 0.64 ± 0.14 (VNIR), 0.62 ± 0.16 (SWIR1), and 0.59 ± 0.17 (SWIR2). In regions where *ω_tree_*(Ω) was at its lowest and thus the ratio of stray light (*b_tree_*DN*_stray_*) to signal from the tree (DN*_tree_*) was high (e.g., on average 61% at 400 nm, 39% at 660 nm, and 52% at 1930 nm), the stray light correction using Eqs. (16), (17) resulted in an average increase of *ω_tree_*(Ω) by 59% at 400 nm, 30% at 660 nm, and 68% at 1930 nm, compared to a simple stray light correction, i.e. *b_tree_* and *b*_WR__*_tree_* set to unity. It also prevented negative *ω_tree_*(Ω) values.

#### 3.6.4 Smoothing of spectra

Similarly to leaf and needle spectra, the processed *ω_tree_*(Ω) spectra were smoothed with Savitzky-Golay filter. This was done separately for each of the three detectors of the spectrometer. Because continuous spectra were required in the modeling, we corrected the data for the observed sensor jumps (Section 3.6.2) by adjusting (multiplying) the *ω_tree_*(Ω) estimates obtained by the SWIR1 and SWIR2 detectors to match those observed by the VNIR detector. It was assumed that VNIR had the smallest errors due to its largest field-of-view (Fig. 2).

#### 3.6.5 *Uncertainties in ω_tree_*(Ω)

Random errors of the measurements were generally small and will be evaluated based on tree-to-tree variability in our results. To evaluate potential systematic errors, we carefully considered all components in Eqs. (14), (16), (17). We estimate that the largest and therefore most relevant systematic errors occur from the PSF correction factor (*f*_WR_*__tree_*/*f_tree_*(Ω) in Eq. (14)), tree silhouette area (*S_tree_* in Eq. (14)), and the stray light correction parameter (*b_tree_* in Eq. (16)). The systematic error in *f*_WR_*__tree_/f_tree_*(Ω) can be at least ± 7% in relative terms, based on the observed ‘sensor jumps’ in the data after correction (Section 3.6.2). *S_tree_*, on the other hand, is sensitive to the thresholding algorithm and camera exposure settings. We estimate that the bias in *S_tree_* can be ± 7%, based on *S_tree_* values computed from images taken with different exposure times. The uncertainty in *b_tree_* is the most difficult to evaluate. Assuming that the error in *b_tree_* equals its standard deviation (0.17), which is a rather conservative estimate and likely an upper limit for the systematic error, error in *ω_tree_*(Ω) would be less than 2% in near-infrared, increasing towards wavelengths where *ω_tree_*(Ω) is low, and peaking to ± 16% at 400 nm, ±10% at 660 nm, and ± 14% at 1930 nm. Based on these estimates, we evaluated the overall uncertainty in the goniometer measurements to be 15–30% in relative terms, being the highest for regions where *ω_tree_*(Ω) is low.

### 3.7 Model evaluation

We evaluated the performance of the model (Eq. (5)) in predicting tree reflectance and directional scattering properties. First, we inverted the values of *p* and *ρ*(Ω) for each view angle using Eq. (6). The value of *p* was constrained to be constant for one tree, because there is no physical reason to assume that *p* would vary depending on view angle. Similarly to Schull et al., 2011, Knyazikhin et al., 2013, we used wavelengths 710–790 nm for the model inversion. In this region, the impact of scattering from leaf surface is negligible (Bousquet et al., 2005) and the directional scattering characteristics of leaves should therefore remain constant. This was supported also by our data, which showed a near-constant reflectance to transmittance ratio of both leaves and needles in this region. Two versions of the model were tested. The first model considered only leaves as plant elements, i.e. leaf albedo spectra were used directly as *ω_leaf_* in Eqs. (5), (6). For each tree, we used leaf albedo averaged over all three samples and over both sides of the sample. The second model included also woody parts, so that *ω_leaf_* equaled weighted average of measured leaf and bark albedo. Relative shares of leaf and woody parts in total plant area were used as weights. Single bark albedo spectrum per species, i.e. average over all three samples measured, was used.

The inverted values of *p* and *ρ*(Ω) were used for calculating *ω_tree_*(Ω) (Eq. (5)). Reflectance of a tree (*ω_tree_*(2*π*)) in both measured and modeled data was computed from the *ω_tree_*(Ω) using the formula

(18) $$\omega_{\mathrm{tree}}\left(2\pi \right)=\frac{2\pi }{12}\sum_{i=1}^{12}\sum_{j=1}^{3}w_{j}\omega_{\mathrm{tree}}\left(\Omega_{\mathrm{ij}}\right)$$

where *i* and *j* are azimuth and zenith angles, and *w_j_* are the weights of Gauss-Legendre integration (see Section 3.3 and Eq. (8) for detailed explanation). The model performance in simulating tree reflectance was evaluated for the region of 400–2300 nm, i.e. noisy UV and shortwave-infrared regions excluded. We computed wavelength- (*λ*) and species- (*sp*) specific bias, root-mean-square-error (RMSE), and standard deviation (SD) as

(19) $$\text{bias}\left(\lambda ,sp\right)=\frac{1}{n}\sum_{i=1}^{n}\left(\omega_{\mathrm{tree},\text{modeled},i}\left(\lambda ,sp\right)-\omega_{\mathrm{tree},\text{measured},i}\left(\lambda ,sp\right)\right)$$

(20) $$\text{RMSE}\left(\lambda ,sp\right)=\sqrt{\frac{1}{n}\sum_{i=1}^{n}{\left(\omega_{\mathrm{tree},\text{modeled},i}\left(\lambda ,sp\right)-\omega_{\mathrm{tree},\text{measured},i}\left(\lambda ,sp\right)\right)}^{2}}$$

and

(21) $$\text{SD}\left(\lambda ,sp\right)=\sqrt{\frac{1}{n}\sum_{i=1}^{n}{\left(\omega_{\mathrm{tree},\text{modeled},i}\left(\lambda ,sp\right)-\omega_{\mathrm{tree},\text{measured},i}\left(\lambda ,sp\right)-bias\left(\lambda ,sp\right)\right)}^{2}}$$

In the above equations, *n* is the number of trees per species, and *ω_tree,_*_modeled_*_,i_*(*λ*,*sp*) and *ω_tree,_*_measured_*_,i_*(*λ*,*sp*) refer to the modeled and measured reflectance for tree *i*. Relative bias, RMSE, and SD (bias-%, RMSE-%, SD-%) were also computed, by normalizing the obtained values by the mean *ω_tree,_*_measured_(*λ*,*sp*).

## 4 Results

### 4.1 Structure and spectra of the tree seedlings

The contribution of woody parts to STAR was generally small. When woody parts were excluded from the total area (*L* in Eq. (7)), tree STAR varied between 0.114 and 0.209 (Table 2). When woody parts were included, on the other hand, tree STAR slightly decreased and varied between 0.099 and 0.200 (Table 2). Spruces deviated from the other species, because their woody to leaf area ratios were relatively large ranging between 0.17 and 0.28. Thus, the STAR values of spruces decreased notably when woody parts were included in computation of total area.

**Table 2:** Structural parameters of the sample trees (min–max).

Parameter	Pine	Spruce	Oak
Height, m	0.38–0.70	0.41–0.70	0.39–0.58
Total leaf area, m^2^	0.20–0.59	0.16–0.48	0.08–0.27
Wood to leaf area ratio	0.05–0.09	0.17–0.28	0.05–0.07
STAR without woody parts*	0.114–0.170	0.118–0.183	0.173–0.209
STAR with woody parts*	0.109–0.158	0.099–0.147	0.162–0.200

* STAR with woody parts means that woody parts were included in calculation of total area. Silhouette areas always included woody parts.

**Fig. 4:**
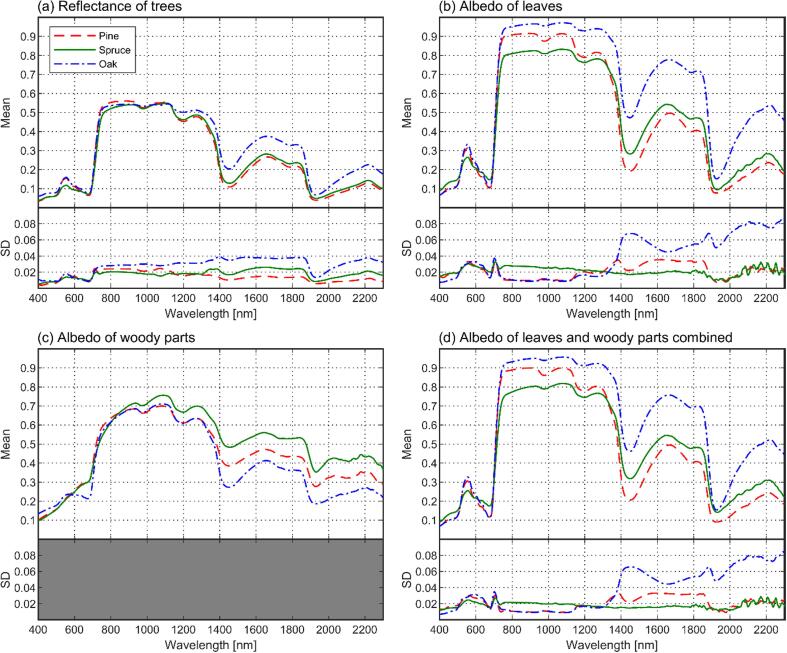
Mean tree reflectance (a), leaf albedo (b), woody part (i.e. bark) albedo (c), and weighted average of the albedo of leaf and woody parts (d) per species. Standard deviations (SD) are given below each figure, representing SD between tree-specific mean spectra. The SD could not be calculated for woody part albedo, because they were only measured for a single sample tree per species.

**Fig. 5:**
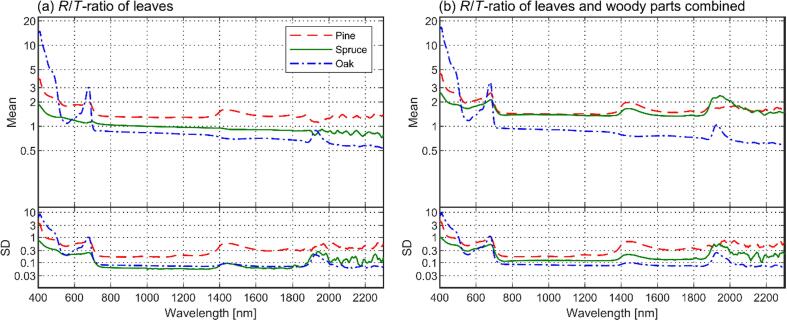
Mean reflectance to transmittance (*R*/*T*) ratios per species, computed from leaf spectra (a) and weighted average of leaf and woody part spectra (b). Standard deviations (SD) are given below each figure, representing SD between tree-specific mean *R*/*T*-ratios. Logarithmic scale is used, because the range of values was large.

The mean tree reflectance was generally similar for all species (Fig. 4a). Oak deviated from the other species the most, because it had clearly the highest reflectance in the shortwave-infrared region (greater than1300 nm). In green wavelengths, spruce showed slightly lower reflectance than the other species, and in blue wavelengths, oak showed slightly larger reflectance compared to the other species. In other wavelength regions, the between-species differences in mean reflectance spectra were relatively small. To support the interpretation of results, we show also albedo of leaves, woody parts, and leaves and woody parts combined (Fig. 4b–d), as well as reflectance to transmittance ratios of leaves and of leaves and woody parts combined (Fig. 5). Notable here is that because spruce had a relatively large fraction of opaque woody parts in its plant area (Table 2), the average reflectance to transmittance ratio of leaves and woody parts combined was notably higher than that of leaves only (Fig. 5). We discuss the optical properties of leaves and woody parts in more detail when interpreting the modeling results (Section 5.1).

### 4.2 Model performance

**Fig. 6:**
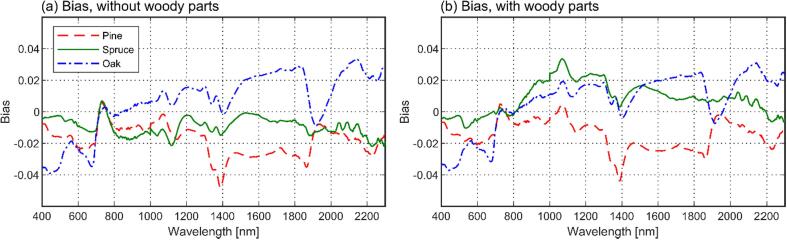
Wavelength- and species-dependent bias in modeled tree reflectance (*ω_tree_*(2*π*)) for models with (a) and without woody parts (b).

**Fig. 7:**
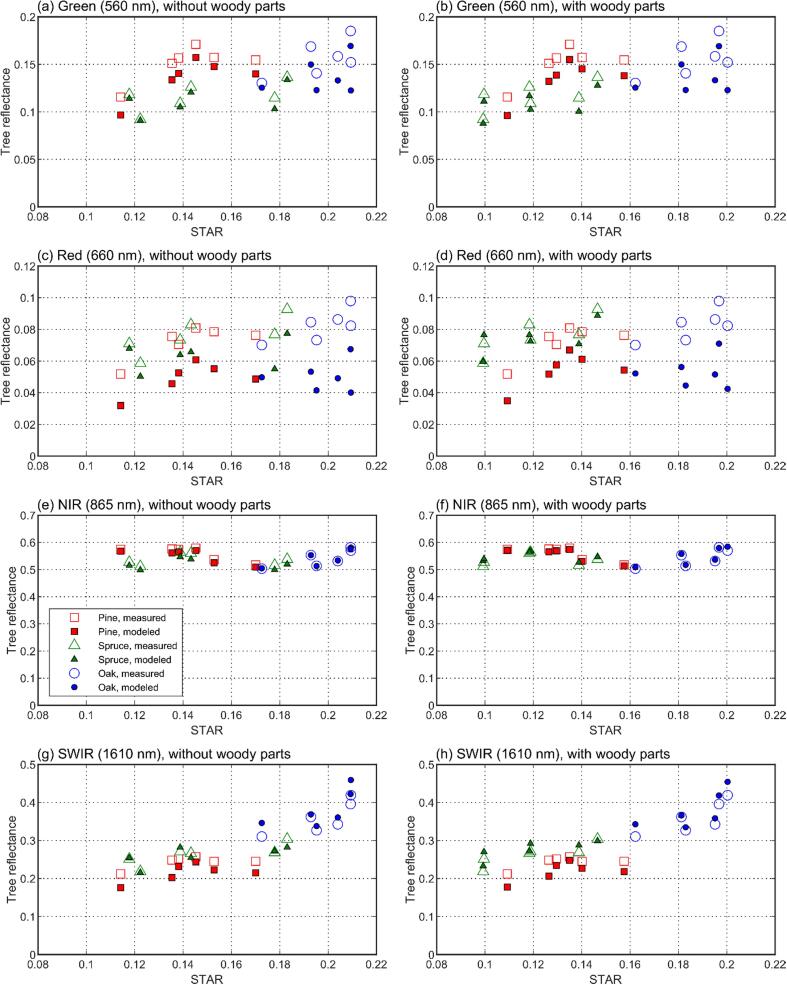
Relations of tree reflectance versus spherically averaged silhouette to total area ratio (STAR) in modeled and measured data. Data are plotted for different wavelengths of the spectrum (top-down). Left column shows results without woody parts, right column shows results including woody parts.

For both models tested (i.e., with or without woody parts), general shapes of the measured and modeled tree reflectance spectra were similar, with species- and wavelength-specific bias varying from −0.048 to 0.034 (Fig. 6). Obviously, the region of 710–790 nm exhibited low bias, because it was used in model fitting. The dependence of tree reflectance on STAR, i.e. the increase in tree reflectance for each tree species at weakly or moderately reflecting wavelengths (e.g. green, red, shortwave-infrared) when the STAR increased, was also reproduced by the model (Fig. 7).

**Fig. 8:**
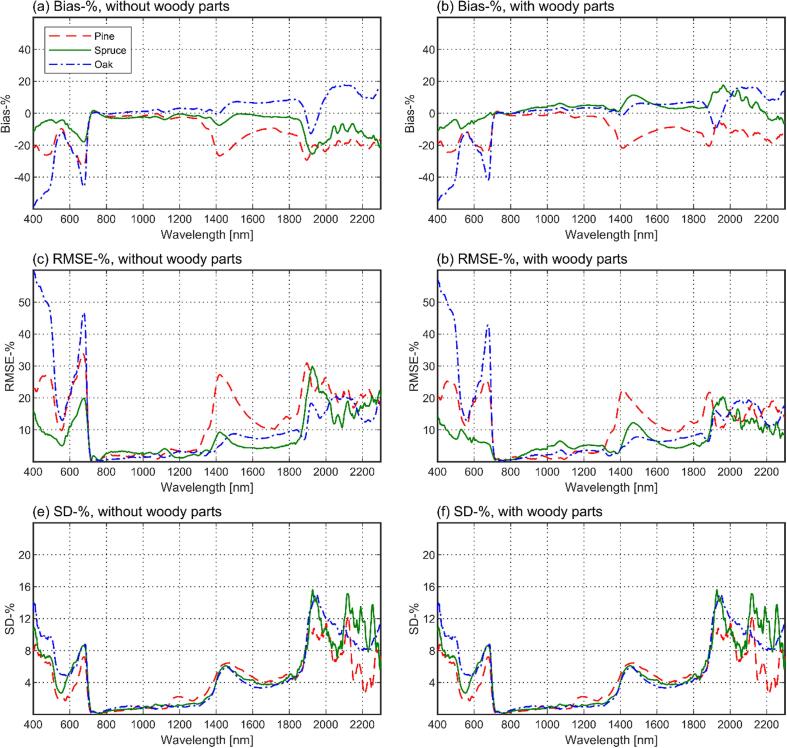
Wavelength- and species-dependent relative bias, RMSE, and SD (top to down) in modeled tree reflectance (*ω_tree_*(2*π*)), separately for models with (left column) and without woody parts (right column).

Next, we looked at the wavelength-dependence of the model performance in more detail by examining the relative bias, RMSE, and SD (Fig. 8). Further, we summarized the mean and maximum bias-% and RMSE-% across all wavelengths, and separately for three wavelength regions (Table 3). These regions exclude the red edge used for model fitting and correspond to the regions of low reflectance in the visible region (400–709 nm), high plateau in the near-infrared (791–1300 nm), and the region with water absorption bands in the shortwave-infrared (1301–2300 nm) (Fig. 4a). It was observed that both models (with or without woody parts) underestimated oak reflectance in the visible region (bias ≥ -58%, RMSE ≤ 60%) and overestimated it in the shortwave-infrared (bias ≤ 18%, RMSE ≤ 21%). Pine reflectance was underestimated in both visible (bias ≥ –33%, RMSE ≤ 34%) and shortwave-infrared (bias ≥ -29%, RMSE ≤ 31%). For spruce, the results varied from underestimation of reflectance in the visible (bias ≥ -18%, RMSE ≤ 20%) and shortwave-infrared (bias ≥ -26%, RMSE ≤ 30%) when woody parts were excluded, to slight underestimation (bias ≥ -12%, RMSE ≤ 15%) and overestimation (bias ≥ 18%, RMSE ≤ 20%) in the same regions, respectively, when the woody parts were included. Model errors in the near-infrared were small for all species (bias from −4% to 6%, RMSE ≤ 7%). Systematic errors dominated in the results, as indicated by the low SD compared to bias, except for the somewhat noisier wavelengths above 1800 nm (Fig. 8).

**Table 3:** Statistics of the wavelength- and species-specific RMSE-% and bias-% of the models with or without woody parts for different wavelength regions. Mean bias-% and RMSE-% were computed as arithmetic means of the wavelength-specific values. “Max. bias (absolute)” means the bias that had the largest absolute value (positive or negative).

	Without woody parts					With woody parts			
	Mean bias	Max. bias (absolute)	Mean RMSE	Max. RMSE		Mean bias	Max. bias (absolute)	Mean RMSE	Max. RMSE
Full wavelength range (400–2300 nm, but excluding 710–790 nm)									
Pine	−14	–33	14	34		−10	−25	12	26
Spruce	−6	−26	9	30		3	18	7	20
Oak	−1	−58	12	60		−1	−55	12	57
Visible region (400–709 nm)									
Pine	–22	–33	22	34		−19	−25	20	26
Spruce	−9	−18	11	20		−6	−12	8	15
Oak	–33	−58	34	60		−31	−55	32	57
Near-infrared (791–1300 nm)									
Pine	−2	−4	2	4		−1	−3	2	3
Spruce	−2	−4	3	4		4	6	4	7
Oak	1	3	2	3		2	4	2	4
Shortwave-infrared (1301–2300 nm)									
Pine	−17	−29	18	31		−13	–22	14	23
Spruce	−8	−26	11	30		6	18	9	20
Oak	7	18	11	21		6	16	10	19

When comparing the results with and without woody parts, it was seen that the inclusion of woody parts improved the model performance for all species in the visible and shortwave-infrared, with maximum RMSE-% decreasing by 3–8 % and 2–10% in these regions, respectively (Table 3). The most notable effect, also seen in Fig. 8a–d, was the reduced underestimation in the red wavelength region. Performance in the NIR was slightly improved for pine (RMSE-% decreased by 1), and slightly reduced for oak and spruce (RMSE-% increased by 3 and 1) (Table 3). However, considering the overall small RMSE-% in the NIR, the decreased performance for spruce and oak is of small importance.

**Fig. 9:**
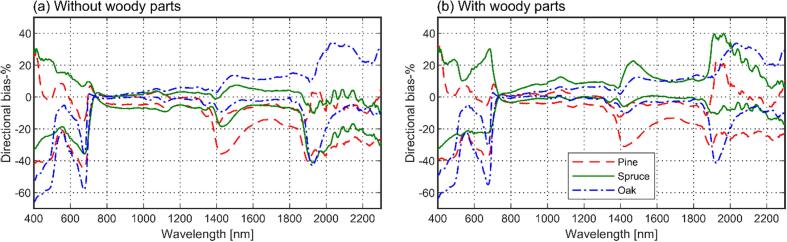
Range of bias-% in modeled *ω_tree_*(Ω), for models without woody parts (a) and with woody parts (b). For each tree species, the lines show the minimum and maximum bias-% among all view directions.

To obtain more insights in the model behavior, we examined the bias for individual view directions, i.e. the bias of *ω_tree_*(Ω) (Fig. 9). It was found that the variation in directional bias was the largest in the visible region, where the difference between minimum and maximum view direction-dependent bias-% was up to 71, 63, and 31 percentage points for pine, spruce, and oak, respectively. In the near-infrared, the respective values were 8, 13, and 8, and in the shortwave-infrared (with noisy region above 1850 nm excluded) they were 25, 25, and 19. Further examination revealed that in the visible wavelength region the model tended to underestimate *ω_tree_*(Ω) of pine and spruce for directions near the illumination, and to produce almost unbiased results or overestimates for the forward scattering side i.e. away from the illumination, which explains the large spread in directional bias-% for these species (Fig. 9).

### 4.3 Model parameters and their relation to tree seedling structure

**Fig. 10:**
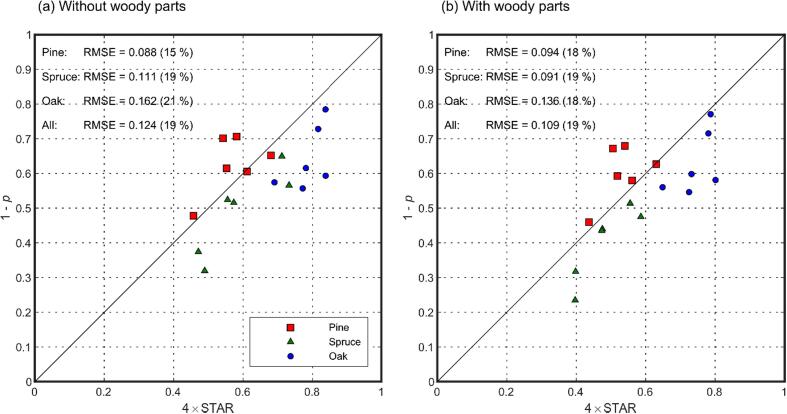
Relation between total escape probability (1 – *p*) and 4 × STAR.

**Fig. 11:**
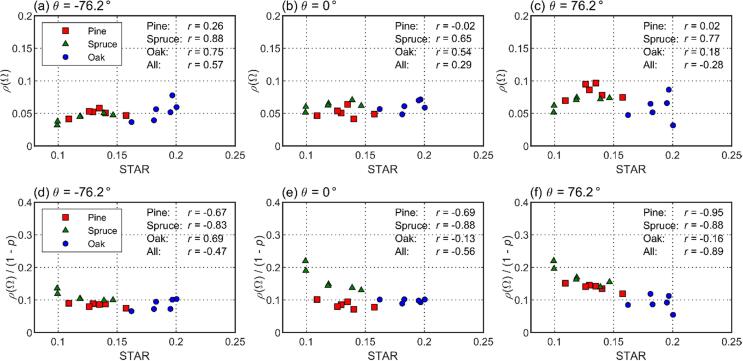
Relations of directional escape probability against STAR (top row), and ratio of directional escape probability to total escape probability against STAR (bottom row) for three view zenith angles (left, middle, and right columns) at azimuth angle of 15°, i.e. close to principal plane. The zenith angles are −76.2° (forward scattering direction), 0° (nadir), and 76.2° (backward scattering direction). Pearson correlation coefficients (*r*) of the relations are given.

Finally, we examined the model parameters and their relations to tree structure. Total escape probability (1 – *p*) followed 4 × STAR quite closely (Fig. 10), which is in accordance with the theory. The RMSE of the relation (1 – *p* vs. 4 × STAR) was 0.124 (19%) when woody parts were ignored, and 0.109 (19%) when woody parts were included (Fig. 10). The values of directional escape probabilities were also mostly physically meaningful, except that mean directional escape probabilities at angles close to the direction of illumination were larger than the theoretical maximum of 0.079: the mean directional escape probability was 0.085 i.e. 8% larger at (*φ* = 15°, *θ* = 48.6°) and 0.088 i.e. 11% larger at (*φ* = -15°, *θ* = 48.6°). The differences are within measurement uncertainties but can be also due to leaves being not fully isotropic, and thus the ‘effective’ escape probability being larger than the theoretical maximum. We also looked at the relations of *ρ*(Ω) and *ρ*(Ω)/(1 - *p*) against STAR (Fig. 11). It was found that *ρ*(Ω) was positively correlated with STAR in view directions on the forward scattering side i.e., the half of the hemisphere that was away from the direction of illumination (Fig. 11a): the mean Pearson correlation coefficient of *ρ*(Ω) vs. STAR relation, using all species and averaged over all view angles on the forward side, was 0.50. The correlation decreased towards the direction of illumination for pine and oak but remained at high level for spruce (Fig. 11b–c), the mean correlation on the backward side being 0.14. The ratio of directional to total escape probability (*ρ*(Ω)/(1 - *p*)) tended to be strongly negatively correlated with STAR on the backward side (Fig. 11f), mean correlation being −0.70. The correlations became weaker towards the forward side but were still mostly negative (Fig. 11d–e), with a mean correlation of −0.53. Despite the above general tendencies, there were large differences between species as seen in Fig. 11, indicating that also other factors than STAR may explain the directionality of scattering.

## 5 Discussion

### 5.1 Performance of the model

For all measured tree species, the modeled tree seedling reflectance spectra followed the shape of the measured spectra reasonably well, with bias ranging between −0.048 and 0.034. The model was also able to reproduce the measured relationship between tree seedling reflectance and STAR. Generally, our study thus confirmed the findings by Rautiainen et al. (2012), who measured Scots pine shoots in a similar goniometer setting and concluded that photon recollision probability can be used for upscaling from needle to shoot albedo. We extended the findings from shoots to tree seedlings, making this the first validation of the photon recollision probability theory at individual tree level.

Our results can be quantitatively evaluated by comparing them to i) uncertainties of satellite reflectance data, ii) similar laboratory measurements made earlier, and iii) to measurement uncertainties in our own experiment. Comparison to satellite data provides a larger context to our results, because ultimately the model would be used for interpretation of satellite data. However, the measured quantities differ, because reflectance factors derived from satellite data are designed for well-defined targets i.e. surfaces. Therefore, we only note that our bias values were somewhat larger than theoretical and empirical uncertainty estimates for satellite-based surface reflectance, which are typically below 0.03 in reflectance factor (Vermote and Kotchenova, 2008, Vermote et al., 2016).

More meaningful is to compare our results to the measurements made by Rautiainen et al. (2012). The RMSE-% of our best-performing model (i.e. the one with woody parts) for Scots pine tree seedlings (up to 26%) were larger than those observed for Scots pine shoots by Rautiainen et al. (2012) (up to ~ 12%). Our study had larger measurement uncertainties, because the sizes of the tree seedlings (height = 0.38–0.7 m) were close to the limit of what can be measured using the goniometer by not violating the balance between geometric, volumetric and isotropic scattering. The models were also delivering different results. We modeled the hemispherical reflectance of trees, instead of full spherical albedo, as done by Rautiainen et al. (2012). Rotating the tree seedlings upside down would have been technically difficult and would have influenced branch orientation. Consequently, we needed to retrieve the spectrally invariant parameters by model inversion (Knyazikhin et al., 2013), because computation of directional escape probabilities by other means is difficult without prior knowledge on the spatial distribution of the foliage. The benefit of our approach was, however, that we obtained information also on how the model performs in predicting directional scattering characteristics of the tree seedlings.

Finally, we compared the model errors to measurement uncertainties in our current study. We first note that the random errors in our measurements were relatively small: for those species and wavelengths which exhibited the largest differences between modeled and measured spectra (i.e. oak in the visible region, and pine in the visible and shortwave-infrared), bias-% clearly exceeded SD-% (Fig. 8). The observed errors were thus mainly systematic. The oak was the only species for which RMSE-% (up to 57% for the best-performing model) notably exceeded the estimated uncertainties in the goniometer measurements (15–30%). Also the RMSE-% for pine (up to 26% for the best-performing model) is close to the upper limit of the estimated uncertainties and indicates some biases in the model. This is especially because the large RMSE-% was observed only for visible and shortwave-infrared regions, but biases in the goniometer measurements, except for those related to stray light correction, should be largely wavelength-independent.

Physical explanation for the somewhat poorer model performance for oak can be attributed to directional scattering characteristics of the foliage. For all species, reflectance to transmittance ratios of foliage were fairly stable for near- and shortwave infrared regions, but increased for the visible wavelength region (Fig. 5). The model bias for oak (Fig. 8a–b) followed a similar but opposite pattern compared to the leaf reflectance to transmittance (*R*/*T*) ratio, i.e. the oak reflectance was underestimated by the model when the *R*/*T* ratio of leaves was high. According to our visual observations, and also supported by earlier measurements for *Quercus petraea* (Farque et al., 2001, Chianucci et al., 2018), the leaf orientation for oak is close to horizontal (planophile). At regions of high *R*/*T* ratio, oak therefore tended to scatter strongly into upper hemisphere, which resulted in the model underestimating tree reflectance. For pine and spruce, dependence of model bias on view direction was such that the model underestimated tree scattering in directions towards the illumination, and overestimated it in the forward scattering side (away from the illumination). Leaf and branch orientation for pine and spruce was difficult to estimate visually, but it might be that, compared to oak, they were more randomly oriented. Therefore, pine and spruce tended to scatter more towards the illumination direction, and less towards the forward direction when the *R*/*T* ratio of needles (or woody parts) was high.

Both pine and spruce behaved similarly, and thus the dependence of model errors on view direction does not explain the negative model bias in simulating hemispherical reflectance for pine. It should be noted that when the needle transmittance is low (as it is in visible region and in the water absorption bands in the shortwave-infrared), the measurements of needle transmittance (Eq. (10)) are prone to errors in the estimation of gap fraction in the needle sample. To demonstrate this, we increased the graylevel threshold value that we used for pine (2 0 2) to the value of 224 that was used by Rautiainen et al. (2012). As a result, the pine needle transmittance and consequently also needle albedo increased. The albedo increase varied from negligible in the near-infrared to 33% at 400 nm. Thus, the observed model errors for pine could be explained by uncertainty in the needle albedo estimates. This highlights the importance of the accuracy of needle optical properties measurements, if we want to increase confidence in model validation results. This is not only true for our results, but also when we validate any radiative transfer model for coniferous forests. However, a general finding from our experiment is that modeled and measured hemispherical reflectance of the tree seedlings agreed within measurement uncertainties, except for the oak in the visible region.

### 5.2 Role of woody parts in model performance

The effect of woody parts on the model performance was small, but generally the inclusion of woody parts improved the match between measured and modeled data. This is in line with earlier studies, in which inclusion of woody parts resulted either in small (Malenovský et al., 2008) or large (Juszak et al., 2014) model improvements, depending on the ratio of wood to leaf areas. Our woody to leaf area ratios fell within the range observed for mature forests, i.e. woody areas up to 30% of total plant area (Stenberg et al., 2003, Verrelst et al., 2010). Based on our results we can conclude that woody parts are an important component to include in forest reflectance simulations and model inversions, as stressed also by other authors (Malenovský et al., 2008, Verrelst et al., 2010, Juszak et al., 2014). However, inclusion of woody parts explained only a relatively small part of the model errors.

### 5.3 Dependence of spectrally invariant parameters on tree seedling structure

Total escape probability (1 – *p*) was linked to 4 × STAR, and thus we empirically proved the theoretical formula (*p* = 1–4 × STAR) that was presented originally for coniferous shoots in Smolander and Stenberg (2003) and was recently shown to be valid also for single trees in a simulation study (Wang et al., 2020). Indirect empirical proof at shoot level was earlier provided by Rautianen et al. (2012), who showed that *p* predicted with the above formula can be used to upscale from needle to shoot albedo. To our knowledge, only that one study has validated the *p* = 1–4 × STAR relation empirically before us. The relation of 1 - *p* to 4 × STAR became slightly stronger when woody parts were included, which further emphasizes the results regarding the small but still important role of woody parts in the modeling of tree reflectance. Both with and without woody parts, however, there were deviations from a perfect linear relationship between 1 - *p* and 4 × STAR, which might be due to both measurement uncertainties and violations of the model assumptions. As discussed in Section 5.1, directional scattering properties of the foliage that differ from isotropic can be an explanation. Oak had the lowest leaf *R*/*T* ratio among the species in the 710–790 nm region used for model inversion, followed by spruce, and then pine (Fig. 5). The under or overestimations of 1 - *p* followed the same order, with 1 - *p* for oak being clearly underestimated, for spruce slightly underestimated, and for pine slightly overestimated (Fig. 10). For spruce, it should be noted that due to the needles being short, their spectra were measured close to their tips where the chlorophyll content might be different from the center of the needle. Thus, the measured spruce needle spectra may not necessarily be fully representative of the average spruce needle spectra, and this might have contributed to the underestimation of 1 - *p* for spruce. Finally, the formula of Smolander and Stenberg (2003) is based on the assumption that the incoming photons are equally distributed on all leaf surfaces, which in real measurements can never be completely fulfilled.

In addition to validating the *p* = 1–4 × STAR formula, our results also gave some indication of how STAR is linked to the directionality of scattering. Higher STAR tended to result in an increased escape probability in the forward directions (away from the illumination), but not as clearly in the backward directions (towards the illumination). Consequently, small STAR (i.e. high clumping and thus more self-shadowing) resulted in larger fraction of the escaped (scattered) radiation being concentrated in directions towards the illumination. Similar empirical results for coniferous (Scots pine) shoots were presented in Rautiainen et al. (2018), and theoretical results for a spherical bush filled with randomly distributed leaves in Dickinson et al. (2008). Thus, STAR might be useful in developing models of directional reflectance of trees and forest. However, it was also evident from our results that the STAR was not the sole predictor of directional escape probabilities, which is in accordance with an earlier study by Schull et al. (2011). They argued that, while 1 - *p* (and therefore also STAR) is linked to forest canopy structure at all hierarchical levels, the ratio *ρ*(Ω)/(1 - *p*) is linked to the macroscale structure of the canopy. This somewhat complicates the possibilities for forward modeling of directional scattering properties of trees or forests. On the other hand, combinations of directional escape probability and 1 - *p* inverted from the measured spectra might reveal species-specific differences in structure, thus enabling to separate species from the hyperspectral data, as was demonstrated by both our results (Fig. 11) and those of Schull et al. (2011).

### 5.4 Implications

The spectral invariants and photon recollision probability theories provide a relatively simple framework for modeling forest canopy reflectance, transmittance, and absorption. Simplicity is preferred in many applications due to low computational cost and intuitive interpretation of the model. For example, concept of spectral invariants has been utilized in producing global maps of leaf area index from data provided by MODIS (Knyazikhin et al., 1998, Myneni et al., 2002), and later also from EPIC multispectral satellite sensors (Yang et al., 2017). These algorithms rely on inversion of reflectance models, because representative combinations of field and satellite data to train statistical methods are difficult to obtain for large areas. Spectral invariant properties are useful in the model inversion, because they reduce the computational cost and facilitate transferability to other sensors (canopy reflectance needs to be modeled only for one wavelength or spectral band), and because they constrain the number of possible solutions to the inversion problem (canopy reflectance at one spectral band is correlated with reflectance at other spectral bands) (Knyazikhin et al., 1998, Myneni et al., 2002, Yang et al., 2017). Another area of application, where spectral invariants or photon recollision probability based models can be used, is hyperspectral remote sensing of leaf biochemical composition, where these models can be used to remove the disturbing effects of forest structure on observed spectra (Knyazikhin et al., 2013). Our results help in increasing confidence in the above mentioned satellite products and algorithms, but they also lead in improved understanding of the assumptions and limitations of the theory. Specifically, as shown by our results and demonstrated earlier also by Panferov et al. (2001), the spectrally invariant parameters are truly spectrally invariant only if the directionality of leaf scattering does not vary over wavelengths. More research should be thus dedicated to evaluating the model assumption about isotropic leaf scattering and its effects on model accuracy depending on the used spectral bands, resolutions, and view-illumination configurations. Ultimately, this would result in improved accuracies of vegetation biophysical variable retrievals from optical remote sensing data.

## Declaration of Competing Interest

The authors declare that they have no known competing financial interests or personal relationships that could have appeared to influence the work reported in this paper.
